# Overall Performance Enhancement of Epoxy Resins Loaded with Non-Covalently Modified Carbon Nanotubes and Graphene Nanosheets

**DOI:** 10.3390/ma19081569

**Published:** 2026-04-14

**Authors:** Marialuigia Raimondo, Liberata Guadagno

**Affiliations:** Department of Industrial Engineering, University of Salerno, Via Giovanni Paolo II, 132, 84084 Fisciano, Italy; lguadagno@unisa.it

**Keywords:** epoxy resin, tunneling atomic force microscopy (TUNA), non-covalent functionalization, carbon nanotubes, graphene nanosheets, thermal properties, morphological analysis, rheology

## Abstract

In this work, we demonstrate that both carbon nanotubes (CNT) and graphene nanosheets (G) were successfully modified by π-stacking interactions with a pyrene derivative (PY), yielding the functionalized nanofillers CNT-PY and G-PY, which were subsequently dispersed within an aeronautical epoxy matrix. This functionalization is highly effective in preserving the remarkable electronic properties of carbon nanotubes and graphene nanosheets. At the same time, the non-covalent functionalization reduces the resin viscosity, enabling a more effective dispersion of the nanofillers. This results in improved rheological behavior and an overall enhancement of the structural performance of the nanocomposites compared to the resin containing unfunctionalized carbon nanofillers (CNT and G). Additional improvements are also observed in electrical properties, self-healing efficiency, and thermal stability. In particular, the samples containing functionalized carbon nanotubes (TBD + 1%CNT-PY) and functionalized graphene nanosheets (TBD + 1%G-PY) exhibit higher conductivities—0.391 S/m and 0.1 S/m, respectively—than the samples loaded with unfunctionalized carbon nanotubes (TBD + 1%CNT) and unfunctionalized graphene nanosheets (TBD + 1%G), which show conductivity values of 0.292 S/m and 4.82 × 10^−3^ S/m, respectively. The functionalized graphene nanosheets (G-PY) display significantly greater thermal stability, with degradation temperatures reaching 670 °C, compared to 310 °C for unfunctionalized ones (G). The functionalized carbon nanotubes (CNT-PY) show a 10% weight loss at 520 °C due to the degradation of the pyrene groups. Significant improvements in the final properties can be achieved when carbon-based nanofillers are homogeneously dispersed in the matrix and the external load is efficiently transferred through strong filler–polymer interfacial interactions, leading to composites with superior characteristics suitable for advanced applications. Tunneling Atomic Force Microscopy (TUNA) highlights the morphological features of the two types of carbon nanofillers, their dispersion within the polymer matrix and the effect of the functionalization on the electrical pathways and conductivity of the samples at both the micro- and nanometer-scale. The measured electrical conductivities are consistent with the electric currents detected at the micro/nanoscale.

## 1. Introduction

Epoxy resins loaded with non-covalently modified carbon nanotubes and graphene nanosheets exhibit considerable improvement in overall performance—mechanical, electrical, and thermal—since the modifiers enhance dispersion and interfacial interactions without compromising the inherent structure of the carbon nanofillers [1,2,3]. Indeed, combining epoxy resins with non-covalently modified carbon nanotubes or graphene nanosheets is one of the most effective strategies for producing high-performance polymer nanocomposites and represents a rich and active research area. The key idea is straightforward: improving the resin by optimizing dispersion, interfacial interactions, and network formation, while preserving the intrinsic properties of carbon nanotubes and graphene nanoparticles. The reasons why this system performs so well arise from several factors, which are clearly outlined below.

The use of non-covalently modified carbon nanotubes and graphene nanoparticles has many advantages:

- Preserved structure and conductivity: Non-covalent functionalization (e.g., π–π stacking with surfactants, polymers, or aromatic molecules) adsorbs onto CNT/graphene surfaces without breaking C–C bonds, thereby maintaining their stiffness, strength, and electrical conductivity [2].
- Better dispersion and reduced agglomeration: Epoxy resins are polar and viscous, while pristine CNTs/graphene tend to bundle via van der Waals forces. Non-covalent modifiers improve compatibility with the epoxy matrix, leading to finer, more uniform dispersion and fewer stress-concentrating agglomerates [1,2,3].
- Improved interfacial adhesion: The modifier layer can interact with the epoxy network (via hydrogen bonding, polar interactions, or mechanical interlocking), creating a more effective load-transfer interface while still leveraging the high modulus of carbon nanotubes and graphene [1].
- Lower percolation thresholds: Non-covalently modified carbon nanotubes or graphene nanosheets efficiently reduce the electrical percolation threshold (EPT), often below 0.1–0.5 wt%, by improving filler dispersion, preventing agglomeration, and preserving high aspect ratios.

Non-covalent modification enables the formation of conductive networks at lower filler loadings while preserving the structural integrity of the matrix. Lowering the rheological percolation threshold of non-covalently modified carbon nanotubes or graphene nanosheets in epoxy is achieved by maximizing filler dispersion and promoting the formation of interconnected networks at reduced filler content. Effective approaches include the use of non-covalent modifiers such as pyrene-based molecules for π–π interactions, optimized high-shear mixing, and in situ polymerization techniques [4,5,6,7]. Integrating non-covalently modified carbon nanotubes or graphene nanosheets into epoxy resins provides a powerful combination of enhanced mechanical, electrical, and thermal performance without compromising the intrinsic properties of the nanofillers. Non-covalent modification is crucial because it preserves the sp^2^ carbon network responsible for the exceptional properties of carbon nanotubes and graphene [1,8,9]. This advantageous integration results in multifunctional nanocomposites characterized by improved mechanical strength, stiffness, thermal stability, and electrical conductivity achieved by exploiting the high aspect ratios and large surface areas of nanocarbons while maintaining their structure and avoiding excessive viscosity increases through non-covalent functionalization [2,10,11,12,13]. Proper functionalization ensures that high nanofiller loadings can be processed without significant viscosity buildup. The multifunctional materials obtained through non-covalent functionalization are particularly relevant for aerospace, automotive, and flexible electronics applications [10,11,14,15]. This study aims to address the limitations associated with poor filler dispersion and agglomeration in epoxy nanocomposites, which typically compromise their mechanical and electrical performance. The non-covalent functionalization of carbon nanotubes and graphene nanosheets is proposed as an effective strategy to improve interfacial interactions and achieve uniform filler distribution, thereby reducing viscosity issues and enhancing the overall performance of the material. Additionally, the objective is to improve the thermal stability and self-healing capabilities of the nanocomposites, making them more suitable for advanced technological applications.

This work proposes a non-covalent functionalization of CNT and G nanofillers that more effectively preserves their structure. The modification is achieved through π–π stacking interactions between the nanofillers and pyrene molecules.

In this work, 1-pyrenebutyric acid (PY) was specifically selected as the non-covalent modifier instead of other aromatic molecules commonly used for CNT/graphene functionalization, because it combines exceptionally strong π–π affinity for graphitic surfaces with chemical features that enhance dispersion, interfacial adhesion, and processing compatibility. In epoxy-based nanocomposites such as those investigated herein, these advantages translate directly into improved microstructure, more efficient stress-transfer networks, and enhanced thermal and mechanical performance.

More specifically, the choice of 1-pyrenebutyric acid arises from its unique ability to couple a highly stable π–π interaction with CNTs and graphene with a polar functional group that is compatible with the epoxy matrix. The pyrene aromatic core ensures stronger and more uniform adsorption than many other commonly used aromatic modifiers, thereby preserving the integrity of the sp^2^ carbon network and promoting a finer, more durable dispersion of the nanofillers. At the same time, the carboxylated butyric chain improves interactions with the epoxy system, fostering a more effective interfacial region and a more homogeneous distribution of the fillers. This combination of strong non-covalent anchoring and chemical compatibility facilitates the formation of continuous, thermally stable, and mechanically efficient conductive and load-transfer networks, with direct impacts on thermal stability, healing kinetics, and the final mechanical properties of the nanocomposite.

These intrinsic characteristics of PY are clearly reflected in the experimental results, particularly in the improved filler dispersion, the faster development of load-transfer networks, and the enhanced thermal stability. Overall, the superior performance observed in our epoxy nanocomposites is closely linked to the deliberate choice of this specific non-covalent modifier.

Consequently, the functionalized CNT and G nanostructures were incorporated into the epoxy matrix to produce functional nanocomposites for structural applications while preserving the electronic structure and properties of the original materials.

The resulting nanocomposites were then morphologically and electrically characterized using Tunneling Atomic Force Microscopy (TUNA), which revealed higher local electric currents in the samples containing functionalized fillers compared to those containing unfunctionalized ones.

TUNA (Tunneling AFM) is an AFM-based technique capable of mapping and quantifying ultra-low electrical currents—down to the sub-picoampere range—at the nanoscale. It has proven particularly effective for carbon-based epoxy nanocomposites containing carbon nanotubes (CNTs), carbon nanofibes (CNFs), graphene nanosheets (GNs), or hybrid CNT/GN networks [4,16,17,18,19,20], where conductive pathways often form at very low filler loadings. The technique overcomes a key limitation of bulk conductivity measurements by revealing where and how local conductive networks develop within the polymer matrix, thus providing nanoscale insight into percolation behavior, network continuity, and localized charge-transport phenomena.

The primary findings indicate that the chemical functionalization of carbon particles inhibits agglomeration during filler dispersion in the viscous resin, thereby streamlining the manufacturing process and ultimately improving the overall performance of the resulting materials. Notably, the non-covalent functionalization enables self-healing behavior, which is completely absent in epoxy samples containing only unfunctionalized nanofiller (CNT and G). In fact, the non-covalently modified nanofillers CNT-PY and G-PY impart self-healing capabilities to the epoxy resin, achieving efficiencies of 30% and 15%, respectively. This demonstrates that the functionalized carbon nanotubes and graphene nanosheets actively participate in the healing mechanism due to their improved dispersion. A well-dispersed nanofiller network is the backbone of many self-healing mechanisms, as it is essential for efficient load transfer, crack bridging, and the formation of conductive or percolated pathways. Interfacial interactions—including π–π interactions—play a key role in enabling healing. These interactions create a dynamic interface capable of reorganizing when a crack forms, allowing the material to “reconnect” after damage.

In summary, non-covalent functionalization enables self-healing because it improves dispersion, preserves the conductive and mechanical properties of the nanofillers, introduces reversible or dynamic interfacial interactions, promotes the formation of percolated networks required for thermal and electrical healing, and enhances polymer mobility at the interface.

## 2. Materials and Methods

In this work, multi-walled carbon nanotubes (labeled as CNT) were used as conductive nanofillers. They exhibit >95% purity, diameters of 10–30 nm, lengths ranging from 100 nm to several micrometers, and consist of 4–11 walls. Graphene nanosheets (labeled as G) were also employed; they feature an exfoliated phase of 60% and were produced through an intercalation–exfoliation process starting from natural graphite with an average particle diameter of 500 µm. More detailed information on CNT and G is provided in the Supplementary Materials.

The 1-Pyrenebutyric acid (PY) was supplied by Sigma-Aldrich (St. Louis, MO, USA). The epoxy matrix (TBD) was formulated by combining the epoxy precursor tetraglycidylmethylenediamine (TGMDA) with the reactive diluent 1–4 butanedioldiglycidyl ether (BDE) in an 80:20 wt% ratio, followed by the addition of the hardener 4,4′-diaminodiphenyl sulfone (DDS) at a stoichiometric amount relative to all epoxy groups.

Epoxy blend and DDS were mixed at 120 °C.

**Figure 1:**
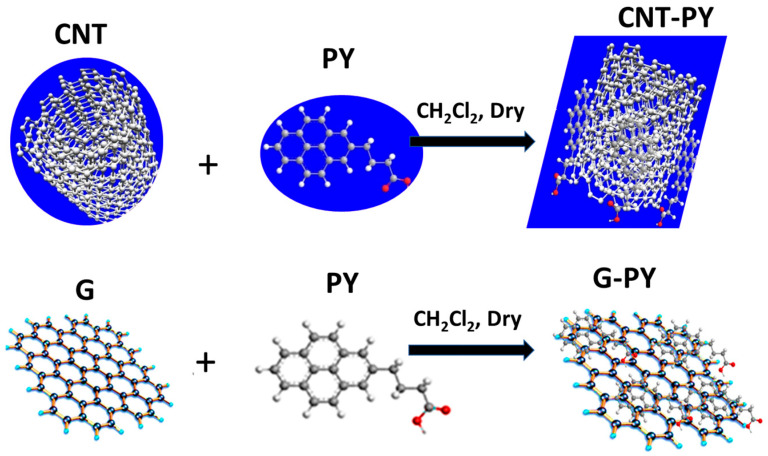
Preparation procedure of the functionalized carbon nanotubes CNT-PY (adapted from our previous paper [7]) and graphene nanosheets G-PY (adapted from our previous paper [4]).

The π–π stacking interactions between PY and CNT, and between PY and G, were achieved by blending PY (≈0.100 g) and the carbon nanofiller (≈1 g) in dry CH_2_Cl_2_ (50 mL) inside a reaction flask (Figure 1). The mixtures were stirred for 2 h at ambient temperature. The products were then filtered, rinsed with CH_2_Cl_2_ (30 mL), and dried overnight under vacuum. The resulting functionalized carbon nanostructures, referred to as CNT-PY and G-PY, weighed approximately 1.080 g.

The carbon nanotubes (CNT and CNT-PY) were incorporated into the matrix at loading concentrations of 0.05, 0.1, 0.32, 0.64, and 1 wt% to produce nanocomposites labeled as TBD + Y%CNT, TBD + Y%CNT-PY. Graphene nanosheets (G and G-PY) were added at 0.025, 0.1, 0.32, 0.5, 1, and 1.8 wt% to produce nanocomposites labeled as TBD + Y%G, TBD + Y%G-PY. In all cases, Y denotes the weight percentage of the respective unfunctionalized and functionalized carbon-based fillers. Ultrasonication was performed for 20 min using a Hielscher model UP200S (24 kHz high-power ultrasonic probe, manufactured by Hielscher Ultrasonics GmbH Oderstr. 53, 14513 Teltow, Germany) to achieve effective dispersion of the carbon nanofillers within the matrix. The epoxy samples were then subjected to a two-step curing procedure: first at 125 °C for one hour, followed by a second stage at 200 °C for three hours.

In this study, several experimental techniques were employed to characterize the developed nanocomposites. Detailed technical information regarding Differential Scanning Calorimetry (DSC), Thermogravimetric analysis (TGA), High-Resolution Transmission Electron Microscopy (HRTEM), Field-Emission Scanning Electron Microscopy (FESEM), rheological measurements, self-healing efficiency evaluation, and electrical characterization using TUNA is provided in the Supplementary Materials.

## 3. Results and Discussion

### 3.1. Morphology of Unfunctionalized and Functionalized Carbon Nanofillers

**Figure 2:**
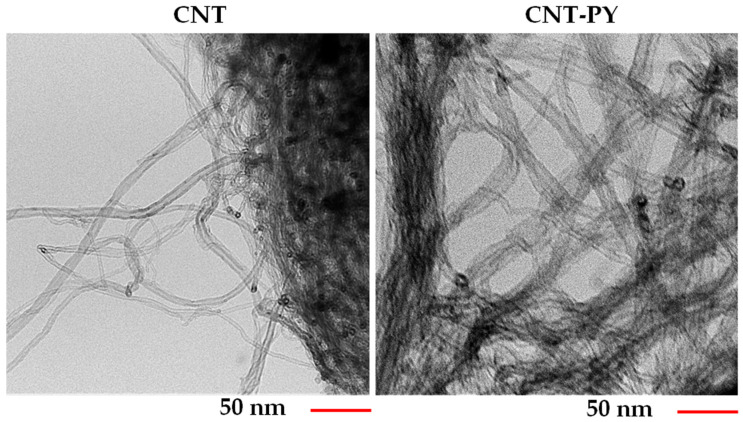
HRTEM images of CNT and CNT-PY.

**Figure 3:**
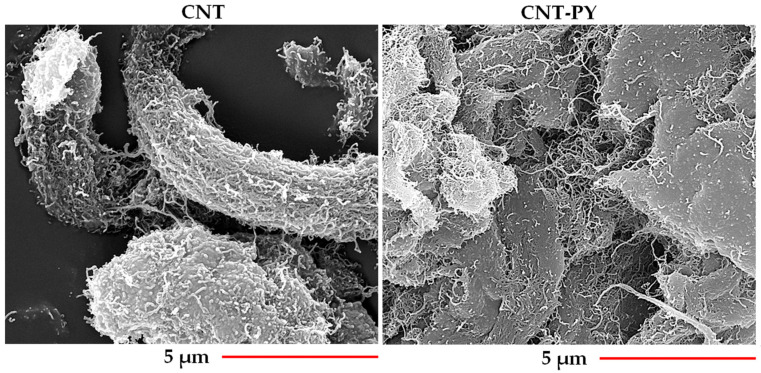
FESEM images of CNT and CNT-PY.

Figure 2 and Figure 3 show HRTEM and FESEM images (captured at the same magnification) of unfunctionalized (CNT) and functionalized (CNT-PY) carbon nanotubes, respectively.

The morphological investigation of functionalized CNT-PY compared with unfunctionalized CNT, conducted through FESEM and HRTEM, demonstrates a markedly improved separation of the functionalized CNT-PY. In contrast, the unfunctionalized carbon nanotubes CNT naturally tend to bundle due to strong van der Waals forces. More specifically, non-covalent functionalization with pyrene enhances separation because it modifies the CNT surface without damaging the sp^2^ structure. The key point is that pristine CNT inherently aggregate due to strong CNT–CNT interactions, whereas non-covalent functionalization disrupts this tendency by reducing inter-nanotube attraction through π–π interactions, steric effects, and, in some cases, mild electrostatic repulsion [21,22,23,24].

**Figure 4:**
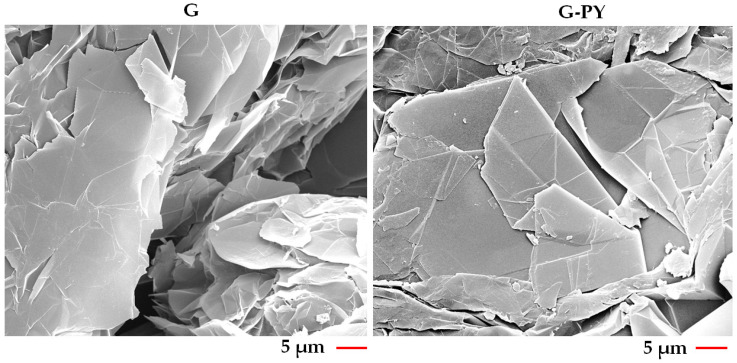
FESEM images of G and G-PY.

Figure 4 shows FESEM images of unfunctionalized (G) and functionalized (G-PY) graphene nanosheets, acquired at the same magnification.

It is evident that both G and G-PY samples exhibit exfoliated graphite sheets with the characteristic fluffy morphology typical of thermally processed graphite. The fluffy, worm-like, highly expanded structure is a direct consequence of the thermal shock used to exfoliate graphite, and both analyzed samples (unfunctionalized and pyrene-functionalized) undergo the same process. Pyrene attaches to graphene through π–π stacking, which does not break C–C bonds, does not introduce defects that would alter the expansion behavior, and does not significantly affect the thermal shock response. Because the graphene lattice remains intact, the exfoliation process proceeds almost identically to the unfunctionalized sample.

Additionally, FESEM images reveal the folds and wrinkles present on the surface of the comparatively large (several-micrometer-scale) and structurally consistent graphene platelets. This observation confirms—consistent with Raman analysis [4]—that layered graphene structure remains intact despite non-covalent functionalization.

### 3.2. DSC Investigation and TGA Analysis

**Figure 5:**
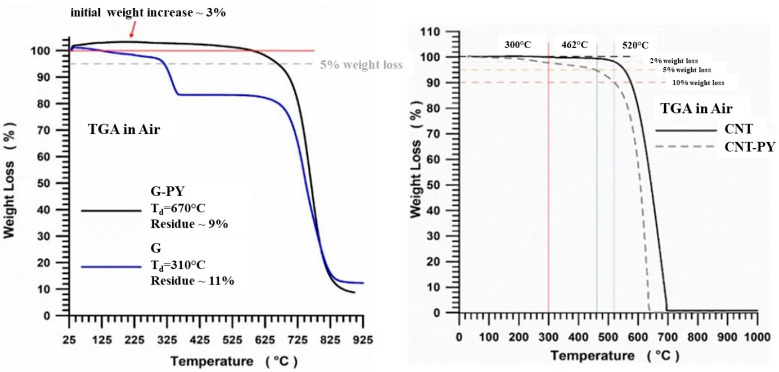
TGA in air of G and G-PY (on the **left**). Adapted from our previous paper [4]; TGA in air of CNT and CNT-PY (on the **right**). Adapted from our previous paper [7].

To facilitate the interpretation of the thermogravimetric results for the systems examined, the TGA profiles—including thermodegradation temperatures (T_d_) and residue amounts—for both the unfunctionalized (G) and non-covalently modified (G-PY) nanofillers, are shown in Figure 5 (left). The TGA curves indicate that the onset of thermodegradation for the functionalized G-PY (black curve) occurs at a significantly higher temperature—approximately 70 °C higher—than that of the unfunctionalized G (blue curve). Specifically, the non-covalent functionalization of graphene by the pyrene derivative markedly enhances the thermal stability of the original nanofiller G, resulting in a substantial improvement in the oxidative thermostability of the composite, as evidenced by the shift of CO_2_ release to higher temperatures. The thermodegradation temperatures (T_d_) are 310 °C for the G sample and 670 °C for the G-PY sample, with corresponding residue values of 9% for G and 11% for G-PY. The significant increase in thermal decomposition temperature—from 310 °C for G to 670 °C for G-PY—is attributed to the non-covalent functionalization with the pyrene derivative, which enhances the oxidative thermostability of the material. This modification suppresses the tendency of graphene to oxidize at lower temperatures, thereby enabling greater thermal resistance. The strong interactions between the PY functional groups and the graphene lattice, mediated by π–π stacking and electron delocalization, contribute to this enhanced stability by effectively passivating defect sites that typically initiate early thermal degradation. As a result, a more ordered and thermally robust carbon framework is formed.

Additionally, the presence of PY moieties promotes char formation and contributes to the development of a protective carbonaceous layer during heating, further delaying decomposition. These combined effects are consistent with the high thermal stability commonly observed in nitrogen-functionalized graphitic materials.

Besides, G-PY nanofillers exhibit an initial weight gain of approximately 3%. Repeated experiments confirmed a small but consistent increase (2–3%). This weight gain is likely attributed to the strong adsorption of nitrogen molecules—linear, relatively small species—on and between the layers of functionalized graphene [4]. The thermogravimetric curves in air for the unmodified CNT and the modified CNT-PY are shown in Figure 5 (right). It is important to note that the unmodified carbon nanotubes (CNT) remain stable up to approximately 520 °C, whereas at this temperature the modified carbon nanotubes (CNT-PY) exhibit a weight loss of about 10 wt%. This weight reduction corresponds precisely to the amount of functional groups attached to the carbon nanotubes, quantified at 10 wt%, and is associated with the thermal decomposition of the pyrene moieties.

**Figure 6:**
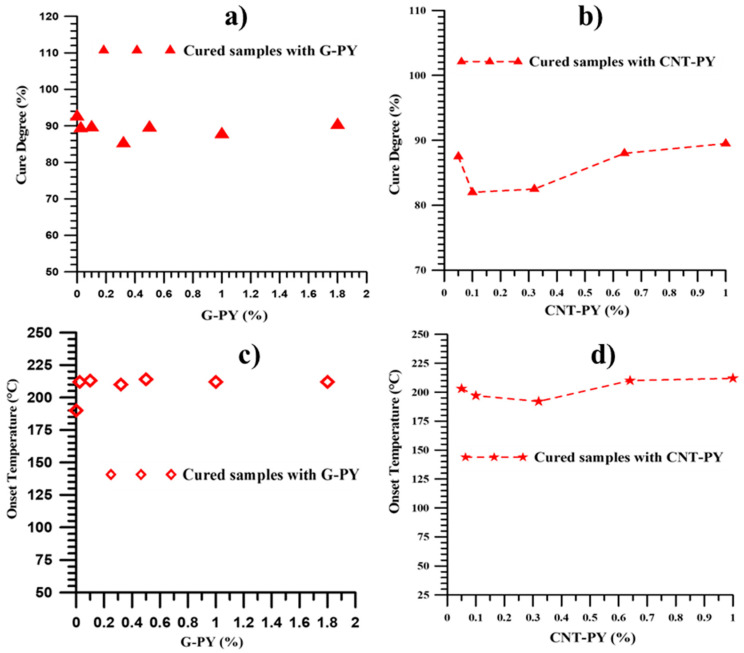
DC values of the statically cured epoxy nanocomposites with different weight percentages of functionalized nanofillers: (**a**) G-PY (adapted from our previous paper [4]) and (**b**) CNT-PY; Cross-link onset temperature trend for the statically cured epoxy nanocomposites with different weight percentages of functionalized nanofillers: (**c**) G-PY (adapted from our previous paper [4]) and (**d**) CNT-PY.

The DC values for the nanocomposites prepared with functionalized G-PY and CNT-PY, cured under constant heating conditions and shown in Figure 6a,b, indicate that all the formulations reach an average curing degree of approximately 90%, which satisfies the requirements of the aviation sector. Figure 6 also illustrates the trend of the cross-linking onset temperatures for the statically cured epoxy samples filled with functionalized G-PY and CNT-PY (Figure 6c,d). All samples cured at 200 °C—undergoing only a “residual cure” stage—exhibit onset temperatures exceeding 180 °C. Furthermore, it is important to note that for all uncured samples containing various weight percentages of the two functionalized nanofillers (G-PY and CNT-PY), the initiation of cross-linking (onset temperature) consistently occurs at approximately 125 °C.

### 3.3. Rheological Properties

**Figure 7:**
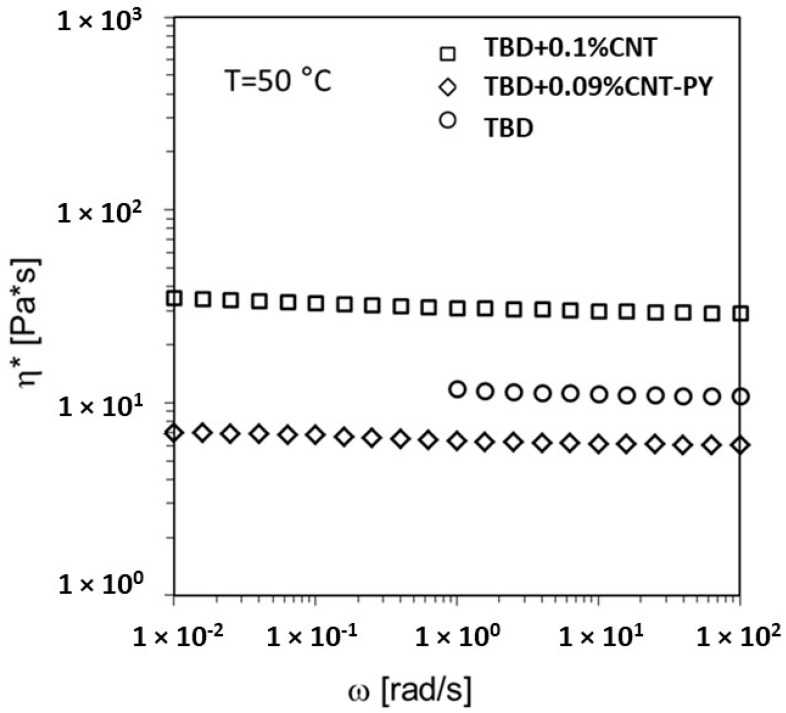
Complex viscosity vs. frequency at T = 50 °C for the TBD epoxy matrix, the TBD + 0.1%CNT and the TBD + 0.09%CNT-PY liquid dispersions. Adapted from our previous paper [7].

The rheological results indicate that the addition of small amounts of carbon nanotubes does not alter the Newtonian behavior of the pure TBD epoxy matrix (Figure 7). Incorporating a small quantity of unmodified CNT into the TBD matrix leads to a marked increase in the viscosity of the uncured epoxy, a trend commonly observed in CNT-based nanocomposites [25]. In contrast, the introduction of functionalized CNT-PY results in a reduction of the complex viscosity of the uncured TBD matrix. Beyond preventing agglomeration during filler dispersion, the non-covalent functionalization plays a key role in lowering the viscosity of the nanocomposites.

**Figure 8:**
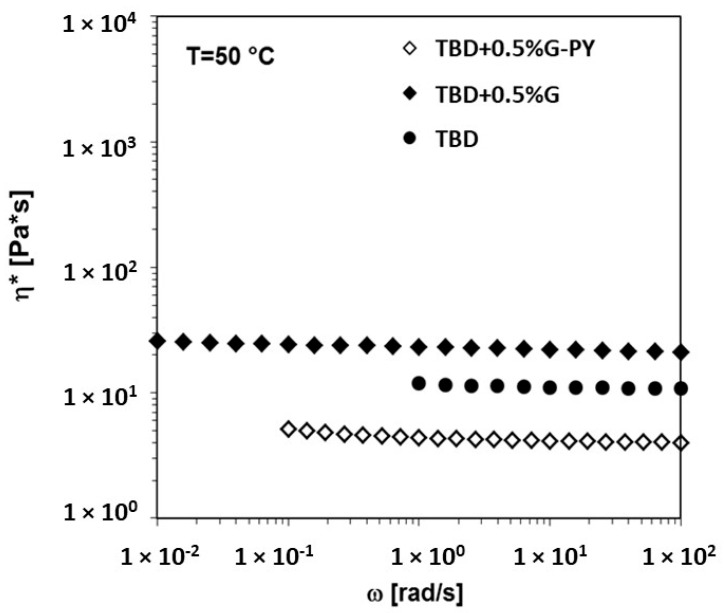
Complex viscosity vs. frequency at T = 50 °C for the TBD epoxy matrix, the TBD + 0.5%G and the TBD + 0.5%G-PY liquid dispersions. Adapted from our previous paper [4].

The complex viscosity (η*) as a function of frequency (ω) for liquid mixtures with 0.5 wt% of unmodified (G) and modified (G-PY) graphene nanoparticles dispersed in the uncured TBD matrix at 50 °C is shown in Figure 8. The addition of unmodified G to the uncured epoxy matrix leads to an increase in viscosity, whereas the incorporation of modified G-PY results in a pronounced reduction of the complex viscosity across the entire frequency range examined. This behavior highlights an important consideration for the industrial processing of epoxy nanocomposites. Pyrene functionalization reduces the complex viscosity because the bulky aromatic groups weaken filler–filler π–π interactions and improve compatibility with the epoxy matrix. This prevents the formation of large agglomerates and decreases polymer–filler friction, allowing epoxy chains to flow more freely around the dispersed nanofillers.

### 3.4. Self-Healing Performance of the Epoxy Composites

By monitoring the evolution of the relative elastic modulus *G_r_* over time, it is possible to determine the efficiency of the healing process. *G_r_* is defined as follows:

$$G_{r}=\frac{G-G_{c}}{G_{p}-G_{c}}$$

where *G* represents the real elastic modulus, *G_c_* denotes the elastic modulus immediately after the crack has developed, and *G_p_* indicates the elastic modulus of the sample prior to crack formation. This procedure enabled us to accurately quantify the material’s ability to recover its rigidity following crack propagation.

**Figure 9:**
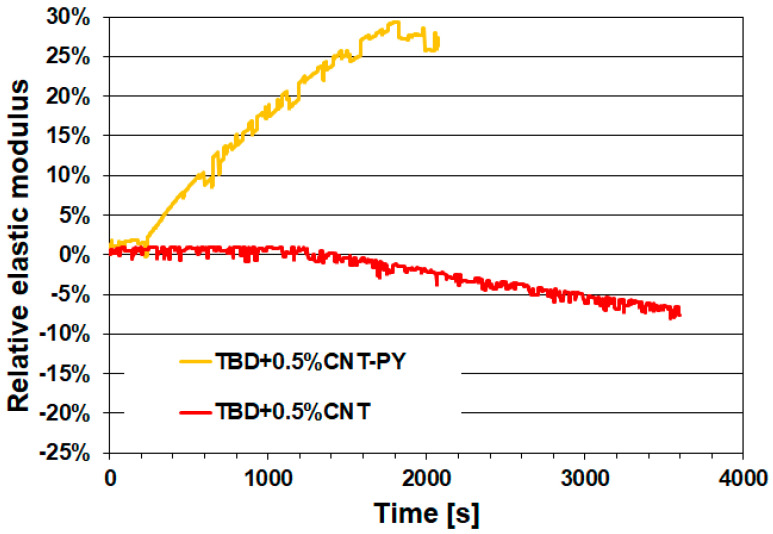
Charts of the relative elastic modulus versus time for the cured epoxy samples TBD + 0.5%CNT-PY and TBD + 0.5%CNT.

**Figure 10:**
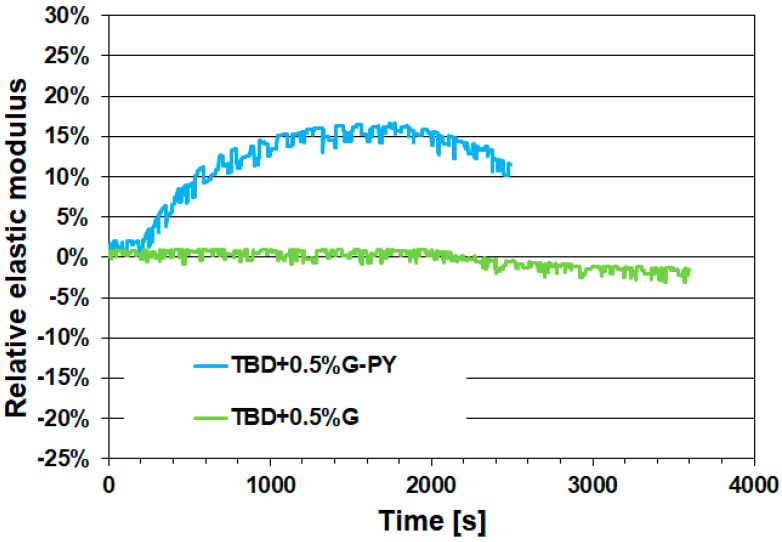
Charts of the relative elastic modulus versus time for the cured epoxy samples TBD + 0.5%G-PY and TBD + 0.5%G.

Below, the healing efficiency values—expressed as the percentage of recovered elastic modulus—for the two epoxy samples containing the non-covalently functionalized carbon nanotubes and graphene nanosheets (TBD + 0.5%CNT-PY and TBD + 0.5%G-PY) are presented in Figure 9 and Figure 10, respectively.

Each functionalized formulation is evaluated against its corresponding sample containing the same weight percentage of unfunctionalized nanofillers (TBD + 0.5%CNT and TBD + 0.5%G). Specifically, Figure 9 and Figure 10 report the evolution of the relative elastic modulus as a function of time for the cured epoxy samples TBD + 0.5%CNT-PY and TBD + 0.5%CNT (Figure 9), as well as TBD + 0.5%G-PY and TBD + 0.5%G (Figure 10).

Figure 9 and Figure 10 indicate that the samples TBD + 0.5%CNT and TBD + 0.5%G exhibit no detectable self-healing efficiency, attesting that the unmodified carbon nanotubes and graphene nanosheets do not impart self-healing capability to the epoxy resin.

Unlike the unmodified carbon nanotubes (CNT) and graphene nanosheets (G), the non-covalently functionalized nanofillers CNT-PY and G-PY are capable of imparting self-healing functionality to the epoxy resin. For the samples TBD + 0.5%CNT-PY and TBD + 0.5%G-PY, healing efficiencies of 30% and 15% were recorded, respectively. The superior performance of the functionalized nanofillers arises from the combination of improved dispersion, preservation of intrinsic nanofiller properties, and enhanced interfacial compatibility with the epoxy matrix. Because the non-covalent functionalization does not restrict polymer mobility, the matrix can reorganize more effectively during the healing process. As a result, CNT-PY and G-PY act as far more efficient promoters of self-healing compared to their unfunctionalized counterparts. This aspect is critical because healing requires polymer chain mobility. Poor interfacial adhesion between unfunctionalized fillers and the polymer matrix often leads to weak, uneven, and irregular interactions. The resulting inefficient stress and load transfer increases local stress concentrations, reduces healing effectiveness, and ultimately compromises the structural integrity of the composite [26,27,28].

When comparing the two nanocomposites TBD + 0.5%CNT-PY and TBD + 0.5%G-PY, the sample containing the functionalized carbon nanotubes (CNT-PY) exhibits the highest efficiency. This result can be attributed to differences in geometry, network formation, and the way each nanofiller interacts with the polymer during the healing process. Epoxy samples incorporating non-covalently functionalized CNT typically show higher healing efficiency because CNT form more effective conductive and stress-transfer networks, disperse more uniformly, promote polymer mobility, bridge cracks effectively, and deliver localized heating with greater effectiveness [29]. Graphene nanosheets, even when non-covalently functionalized, cannot match the network connectivity or healing-activation efficiency of CNTs at comparable loadings [30]. Due to their rigid, planar structure, graphene sheets tend to restrict polymer mobility in their vicinity. Their large surface area can immobilize portions of the matrix, reducing the chain mobility required for efficient healing [31].

### 3.5. Morphology and Nanoscale Electrical Property Mapping by Means of TUNA of the Epoxy Nanocomposites

Tunneling Atomic Force Microscopy (TUNA) is a powerful technique for investigating both the morphology and the nanoscale electrical properties in the epoxy nanocomposites loaded with carbon-based nanofillers, such as carbon nanotubes and graphene nanosheets. Epoxy systems containing carbon nanofillers operate near the threshold of electrical percolation: conductive pathways continuously form, break, reconnect, and reorganize at the nanoscale. Conventional characterization tools cannot directly visualize these dynamic, sub-percolative networks—TUNA can. More specifically, TUNA is capable of detecting currents down to the femtoampere range, which is essential because CNTs or graphene sheets may be separated by extremely thin epoxy layers. In such cases, charge transport occurs predominantly through tunneling rather than direct physical contact, and the earliest stages of percolation generate extremely weak currents. TUNA therefore provides a unique ability to correlate morphology with electrical behavior. Because TUNA can simultaneously acquire topography and current maps, it allows direct comparison of filler dispersion, the presence of agglomerates, interfacial regions, and the formation of conductive pathways. This correlation lies at the core of structure–property relationships in nanocomposites.

For example, a CNT bundle may appear large in the topographical image but exhibit no measurable current if it is poorly connected to the surrounding network. Conversely, a thin graphene sheet may display a strong current signal if it bridges two conductive regions. Such nanoscale insights are inaccessible through bulk electrical measurements.

Because TUNA is inherently a local technique, careful sampling is essential to ensure meaningful and representative results. For each sample, TUNA maps were acquired over five distinct areas, using multiple scan sizes (typically 1 × 1, 2 × 2, 5 × 5, 10 × 10, 15 × 15, and 20 × 20 µm^2^), selected from spatially separated regions of the surface to avoid bias from any single location. The trends reported in the manuscript were consistently observed across all probed regions, with variations remaining within the expected experimental scatter. Although TUNA provides localized measurements, the reproducibility of the results across multiple independent areas supports the statistical representativeness of the observed behavior.

**Figure 11:**
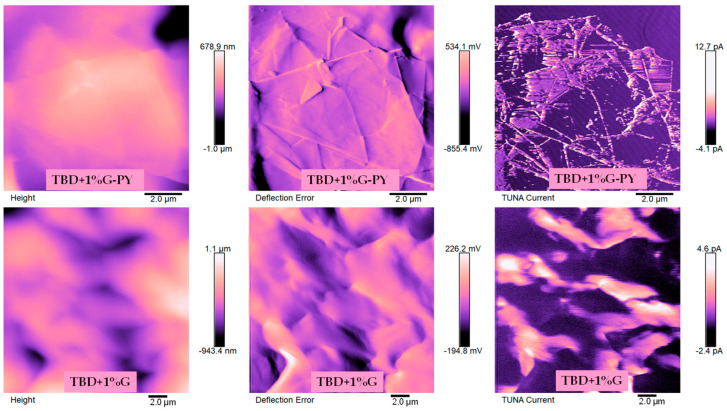
TUNA pictures of TBD + 1%G-PY and TBD + 1%G.

**Figure 12:**
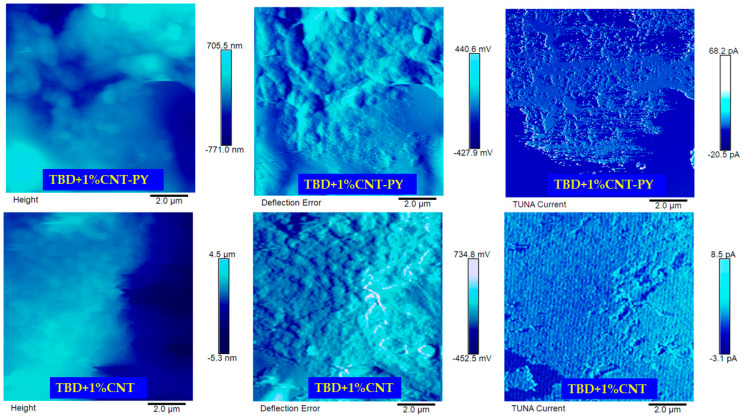
TUNA pictures of TBD + 1%CNT-PY and TBD + 1%CNT.

Figure 11 and Figure 12 show the TUNA images—Height, Deflection Error and TUNA Current—of the samples TBD + 1%G-PY, TBD + 1%G and TBD + 1%CNT-PY, TBD + 1%CNT, respectively. All three images were acquired concurrently at the same magnification to enable a direct and meaningful comparison among the analyzed samples. These three signals complement one another, and understanding their individual roles is essential for correctly interpreting AFM/TUNA data. Their combined analysis is precisely what makes TUNA such a powerful technique for studying epoxy nanocomposites. The Height image provides information on physical morphology and is crucial for locating nanofillers, identifying surface features, and assessing dispersion quality. More precisely, the Height image shows: (a) surface topography—peaks, valleys, roughness, and overall morphology of the epoxy matrix and embedded nanofillers; (b) distribution of nanofillers—CNTs and graphene sheets often appear as raised or elongated features because they protrude slightly from the matrix or create local height variations; (c) quality of dispersion—agglomerates, clusters, or well-dispersed individual nanofillers can be identified; (d) surface defects or irregularities—cracks, voids, or resin-rich regions. The Deflection Error Image shows: (a) material contrast—differences in stiffness or mechanical response between epoxy and carbon nanofillers; (b) sharp edges and boundaries—CNTs, graphene sheets, and agglomerates stand out more clearly; (c) subsurface or partially embedded fillers—because changes in tip–sample interaction appear even when height differences are small; (d) interfacial regions that may not be obvious in the Height image—variations in adhesion or modulus at the filler–matrix interface. In systems such as CNT- or graphene-filled epoxy, the Deflection Error is particularly useful because it allows one to: distinguish individual nanofillers from the surrounding matrix, identify agglomerates or regions with poor dispersion, visualize interfacial zones that influence both mechanical and electrical behavior. The Deflection Error signal enhances edge contrast and highlights subtle morphological variations that may not be evident in the Height image alone, making it a key component in interpreting AFM/TUNA datasets. The TUNA Current image highlights the nanoscale electrical pathways within the composite. By mapping local current flow, it distinguishes electrically active fillers from those that are isolated or poorly connected within the epoxy matrix. This kind of image visualizes percolation networks, shows how dispersion quality affects conductivity and helps correlate morphology (from topography) with electrical behavior. TUNA images provide clear insight into the effects of non-covalent functionalization on carbon nanotubes and graphene nanosheets within epoxy composites. By simultaneously mapping morphology and local electrical activity, TUNA reveals how functionalization influences filler dispersion, interfacial behavior, and the formation of conductive pathways. Furthermore, TUNA imaging offers direct evidence of the improvements in electric currents achieved through non-covalent functionalization of carbon nanotubes and graphene nanosheets in epoxy composites. This technique highlights how functionalization promotes better filler dispersion, interfacial compatibility, and the formation of more efficient conductive networks.

This type of functionalization can enhance the ability of the carbon nanoparticles to interact with the host matrix at the interfacial nanodomains, where they tightly bind to form a large conductive network (see samples TBD + 1%CNT-PY and TBD + 1%G-PY) responsible for detecting higher electric currents values (see color sidebar on the right in the TUNA Current image of Figure 11 and Figure 12) than those recorded for samples loaded with the same percentage of unfunctionalized fillers (see samples TBD + 1%CNT and TBD + 1%G). By carefully observing the TUNA images, it is possible to discriminate the intrinsic morphological details of the two different types of nanofillers effectively dispersed in the epoxy matrix. Specifically, the functionalized G-PY’s surface resembles a drapery’s corrugated texture (see the Height, Deflection Error, and TUNA Current photos at the top of Figure 11). The sidebar, which links the colors to the various recorded current values and provides an efficient mapping of the nanodomains with areas of high current density where the graphene nanolayers appear to be nearly “fused” with the epoxy resin, makes the accentuated contrast in the brilliance of the colors clearly visible in the TUNA Current images of Figure 11. The possibility of measuring significant electrical currents ranging from −4.1 pA to 12.7 pA for the TBD + 1%G-PY sample highlights the high electrical conductivity value of 0.1 S/m and the success of the non-covalent functionalization carried out by choosing the PY compound. For the G-PY nanoloaded sample, the presence of strong interfacial interconnections between the graphene nanosheets and the host matrix is observed, giving rise to a solid conductive network extended over the entire area investigated. Also, for the TBD + 1%G sample, the presence of the unfunctionalized G-PY graphene nanosheets uniformly dispersed in the matrix ensures a conductive surface on which it is possible to detect electrical currents ranging from −2.4 pA to 4.6 pA (see TUNA Current image at the bottom of Figure 11). These values, although high, are lower than those obtained for the TBD + 1%G-PY sample and agree well with the electrical conductivity value of 4.82 × 10^−3^ S/m measured for the TBD + 1%G sample, which is therefore less conductive than the PY-functionalized graphene-nanoparticle-filled sample.

It is worth noting that the lower electrical conductivity value recorded for the TBD + 1%G sample is reflected in the TUNA image, where the weaker interfacial interactions translate into electrically conductive paths that exhibit some signs of discontinuity on the investigated surface.

A direct comparison of these conductivity values reveals that the non-covalent functionalization of graphene nanosheets with pyrene derivative molecules to create G-PY not only maintains the electrical properties of the nanocomposites but also results in two orders of magnitude enhancement in electrical conductivity compared to the resin containing the same 1 wt% of unfunctionalized G. The 1 wt% of G-PY was selected because it exceeds the EPT, which ranges from 0.025 to 0.1 wt% for G-based nanocomposites [18]. The unfilled epoxy matrix (TBD) has volume conductivity at room temperature of approximately 6.00 × 10^−14^ S/m. The improvement in electrical conductivity is probably linked to a more effective dispersion of the graphene nanosheets and the characteristics of the functionalizing PY compound.

The uniform dispersion of the functionalized CNT-PY inside the resin is clearly detectable in all the TUNA images of the sample TBD + 1%CNT-PY (Figure 12 on the top). The presence of the functional groups prevents CNTs from agglomerating through π–π interactions, leading to strong interfacial interactions between carbon nanotubes and the epoxy matrix. HRTEM and FESEM analyses (Figure 2 and Figure 3) show a loss of effective lower separation of the unfunctionalized carbon nanotubes compared to those functionalized with PY. This also emerges from a comparison between the TUNA images of the two different samples, TBD + 1%CNT-PY and TBD + 1%CNT. In particular, in the TUNA Current image of the TBD + 1%CNT sample, the carbon nanotubes appear intimately interconnected, forming a continuous blanket that covers the entire analyzed surface. In a few places, some entanglements of carbon nanotubes appear due to their tendency to agglomerate when unfunctionalized. Nonetheless, it is evident that both nanocomposites contain a continuous network of carbon nanotubes, which is what gives the samples their electrical conductivity which is 0.391 S/m for the nanocomposite TBD + 1%CNT-PY and 0.292 S/m for the nanocomposite TBD + 1%CNT. This proves that the sample containing functionalized carbon nanotubes (TBD + 1%CNT-PY) is more conductive than the sample loaded with unfunctionalized carbon nanotubes (TBD + 1%CNT-PY). This result is in line with the electric currents measured at the micro/nano scale which are higher for sample A being between −20.5 pA and 68.2 pA compared to those detected for sample B which presents values ranging from −3.1 pA to 8.5 pA. The 1 wt% of CNT-PY was selected for comparison with unfunctionalized CNT since the values of the electrical conductivity displayed by the CNT-based nanocomposites for this nanofiller quantity were above the EPT, namely [0.1–0.32] wt% [7].

The current profile makes it possible to identify areas with varying brightness that correspond to various current values. The most conductive regions of the samples, where the presence of carbon nanofillers guarantees the effective transport of the electrical properties on the surface of the resin, exhibit distinct chromatic variations, which are indicative of the well-defined conductive pathways caused by the non-covalent functionalization closely related to the effective dispersion of the conductive nanofillers.

The morphologies derived from TUNA analysis alongside the electric current values obtained at the micro/nanometer scale for the four nanocomposites analyzed, TBD + 1%G-PY, TBD + 1%G, TBD + 1%CNT-PY, and TBD + 1%CNT, allow us to demonstrate the effectiveness of non-covalent functionalization and their improved electrical performance.

## 4. Conclusions

This work explores the functionalization of carbon nanotubes (CNT) and graphene nanosheets (G) using π-stacking interactions with a pyrene derivative (PY), resulting in modified nanofillers G-PY and CNT-PY, thus evaluating the effects of the functionalization on the overall performance enhancement of multifunctional nanocomposites.

The performed functionalization improves the electrical, thermal, mechanical, and rheological properties of the nanocomposites compared to the resin loaded with both unfunctionalized CNT and G. The main results are summarized below:

- The non-covalent modification does not disrupt the layered structure of G and allows for better separation of CNT-PY compared to untreated CNT.
- Thermal analysis shows that this non-covalent modification significantly increases the thermal stability of unfunctionalized G by about 70 °C and enhances the oxidative thermostability of the composites. Pristine CNT remains stable up to about 520 °C, while modified CNT-PY experiences about 10 wt% weight loss at this temperature due to pyrene degradation.
- The degree of cure (DC) values of the functionalized G-PY and CNT-PY nanocomposites cured under isothermal heating are about 90%, meeting aviation industry standards. All samples cured at 200 °C show cross-link onset temperatures above 180 °C, while uncured samples begin cross-linking at around 125 °C.
- A significant decrease in viscosity occurs with functionalized graphene (G-PY) and functionalized carbon nanotubes (CNT), thus impacting industrial preparation processes.
- Non-covalently modified carbon nanotubes (CNT-PY) and graphene (G-PY) enhance the self-healing properties of epoxy resin, achieving efficiencies of 30% and 15%, respectively. Their superior dispersion and interfacial compatibility facilitate better healing compared to unmodified versions.
- The nanocomposite with functionalized carbon nanotubes (CNT-PY) showed the highest efficiency compared to the one with graphene (G-PY). This is due to better network formation and interactions during healing, as CNTs improve conductivity, crack bridging, and localized heating. Graphene’s rigidity limits polymer movement, reducing healing efficiency.
- The TUNA analysis shows that the TBD + 1%G-PY sample exhibits significant electric currents ranging from −4.1 pA to 12.7 pA, which confirms the high conductivity of 0.1 S/m. The electric currents measured for the sample TBD + 1%CNT-PY range from −20. 5 pA to 68. 2 pA and are well correlated with the high conductivity of 0. 391 S/m. The good electrical performance at the nanoscale is due to effective non-covalent functionalization with PY.
- These results show that non-covalently modified G-PY and CNT-PY can be effectively used to create functional nanocomposites for structural applications while preserving the electronic structure and properties of the original materials.

Functionalized nanocomposites hold promise for advanced structural applications but may present limitations in terms of long-term stability and compatibility with other materials. Future research could focus on optimizing filler dispersion and exploring other chemical modifications to further improve performance in aeronautical and industrial contexts. The most interesting challenge lies in designing stable and multifunctional interfaces without sacrificing the conductivity and dispersion of the nanofillers.

## Data Availability

The original contributions presented in this study are included in the article. Further inquiries can be directed to the corresponding author.

## Supplementary Materials

The following supporting information can be downloaded at https://www.mdpi.com/article/10.3390/ma19081569/s1, Figure S1: Test geometry adapted for the healing tests (see on the left); epoxy specimens before and after healing experiments (see on the right).
