# Supplementary material for: Overall Performance Enhancement of Epoxy Resins Loaded with Non-Covalently Modified Carbon Nanotubes and Graphene Nanosheets
Source: Materials (Basel). 2026 Apr 14;19(8):1569. doi: 10.3390/ma19081569 (PMC13117810; doi:10.3390/ma19081569)
Supplement: Supplementary file 1 [file materials-19-01569-s001.zip › materials-4177756-supplementary.pdf]

Supplementary electronic materials

Overall performance enhancement of epoxy resins loaded with non-covalently modified carbon nanotubes and graphene nanosheets

Marialuigia Raimondo * and Liberata Guadagno

Department of Industrial Engineering, University of Salerno, Via Giovanni Paolo II, 132, 84084 Fisciano, Italy; lguadagno@unisa.it

* Correspondence: mraimondo@unisa.it

Experimental section

The outer diameter and the length of the multi-walled carbon nanotubes (3100 Grade - Nanocyl S.A, Sambreville, Belgium), here named as CNT, range from 10 to 30 and from 100 to 1000 nm, respectively. Brunauer–Emmett–Teller method has allowed us to determine the specific surface area of CNT whose value is about 250–300 m^2/g. The thermogravimetric analysis showed a carbon purity greater than 95%.

The conductive graphene nanosheets (G) were created by exfoliating natural graphite known for its large surface area with a mean diameter of 500 μm (Asbury graphite grade 3759, Asbury Carbons, New Jersey (NJ)), through conventional acid intercalation followed by a rapid treatment at elevated temperature (900°C) in a reactor to achieve the expansion of graphene layer spacing [1]. The proportion of the exfoliated phase of nanofiller G is 60%. Its uniqueness lies in possessing a very high concentration (10 wt%) of carboxylated groups found at the edges of the graphene sheet or graphitic blocks, which contribute to the development of self-assembled structures that enhance the mechanical and electrical properties of the final composites [1]

The G nanoparticles within the resin comprise tiny multilayer stacks of graphene, varying from 1 to approximately 16 nm in thickness, and having diameters from sub-micrometer to several tens of micrometers. The space between graphitic layers is approximately 5 to 10 nm. G sample includes graphitic blocks made up of several layers ranging from 5 to 29 [1].

A Mettler DSC 822 differential scanning calorimeter (Mettler-Toledo, Novate Milanese, Italy) in a flowing nitrogen atmosphere was used to carry out thermal characterization between 0 and 300 °C with a scan rate of 10 °C min^{-1}. The Cure Degree (DC) of the samples was evaluated using the calorimetric data [2].

A Mettler TGA/SDTA 851 thermobalance (Mettler-Toledo, Novate Milanese, Italy) was used to carry out the thermogravimetric analysis TGA. The temperature range between 0°C and 1000°C at a 10°C/min heating rate under both nitrogen and air flows was used to heat the formulated samples.

High-Resolution Transmission Electron Microscopy (HRTEM) characterization of CNT and CNT-PY nanofillers was performed on a Jeol 2010 microscope operating at 200 kV.

Micrographs of the unfunctionalized and functionalized carbon nanotubes and graphene nanosheets were obtained using Field Emission Scanning Electron Microscope (FESEM) (mod. LEO 1525, Carl Zeiss SMT AG, Oberkochen, Germany). All samples were placed on a carbon tab previously stuck to an aluminum stub (Agar Scientific, Stansted, UK) and were covered with a 250 Å-thick gold film using a sputter coater (Agar mod. 108 A).

The rheological measurements in the liquid state, before curing, were carried out on a Physica MCR 301 (Anton Paar) rotational rheometer equipped with a parallel plate geometry (50 mm diameter, 1 mm gap). The measurements were performed on the ternary TGMDA-BDE-DDS mixture (TBD), dispersions of the unfunctionalized CNTs and graphene nanosheets G, and functionalized CNTs and functionalized graphene nanosheets G in the TBD matrix.

To assess the crack-healing efficiency of the composite materials, experiments were performed by using a dynamic mechanical thermo-analyzer (TA instrument-DMA 2980). Solid samples with dimensions 3 × 10 × 35 mm^3 were tested by applying a variable flexural deformation in single cantilever mode. A V-shaped starter notch, 1 mm deep and 2 mm wide, was machined close to the sample extremity as shown in Figure S1. This geometry allowed for controlled guidance of subsequent crack propagation.

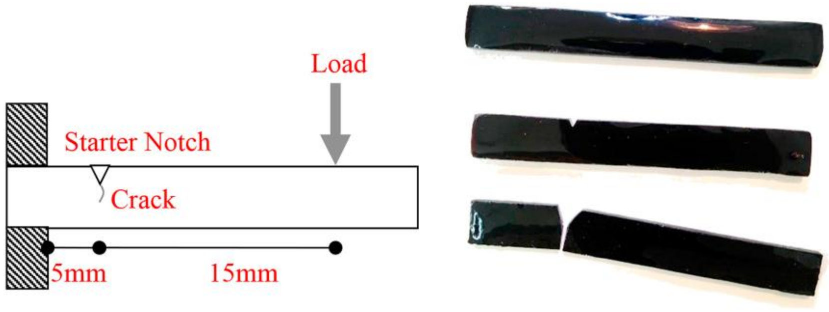

Figure S1. Test geometry adapted for the healing tests (see on the left); epoxy specimens before and after healing experiments (see on the right).

The test procedure combines three different steps. In the first step, the notched sample was analyzed under the dynamic flexural deformations for about 300 s in order to determine the pristine elastic modulus. After that (second step), the oscillation was stopped and a sharp pre-crack was created in the samples by gently tapping a fresh razor blade into a machined starter notch. An impulsive load of about 25 N was immediately applied to the specimen in order to produce crack propagation along the virgin crack plane. At this point (third step), the dynamic monitoring of the sample (healing phase) was conducted at constant temperature of 130°C for 1 h, conditions selected to activate segmental matrix mobility and promote recombination of the fracture surfaces. Throughout the isothermal period, the elastic modulus was continuously monitored by applying a displacement amplitude of 0.1% and a frequency of 1 Hz, to avoid introducing further damage and to monitor the evolution of the mechanical recovery in real time. Several test conditions were investigated in order to obtain a reasonable picture of the amount and time of recovery without influencing the healing mechanisms.

Information on topography and local nanoelectrical current of the multifunctional nanocomposites was obtained by TUNA technique operating in contact mode and using platinum-coated probes with nominal spring constants of 35 N m^{-1} and electrically conductive tip of 20 nm.

The TUNA module measures ultra-low currents (<1 pA) ranging from 80 fA to 120 pA circulating through the conductive tip to the investigated samples kept at a fixed DC bias. In this work, we used a DC sample bias from 1 V to 2 V.

A linear current amplifier with a range of 60-120 fA detects the resulting current passing through the samples. In this way, the sample's topography and current are measured at the same time, activating direct correlation of a sample location with its electrical properties.

It is worth noting that notably sensitive current measurements are allowed due to the noise level of the TUNA module commonly of 50 fA.

Highest resolution current mapping of the nanocomposites was obtained with the current sensitivity of the TUNA module which selects the gain referring to the output voltage of the TUNA sensor set to 1pA/V, corresponding to the gain of 10 12 , scan rate of 0.500 Hz s^{-1}, number of pixels in X and Y (samples/line) set to 512.

The detected areas of the analyzed samples are representative of the entire multifunctional nanocomposites because, in order to obtain electrical measurements at nanoscale level with indisputable repeatability and reproducibility, a cantilever with a sharp tip has scanned on different areas over a sample surface so that each TUNA image reported in the manuscript was captured after verifying that the electrical response was the same at least five various scanned points.

It is worth noting that, generally, it is not enough that the tip is in contact with a conductive material but electrical contacts to the ground ensured by silver paste are also essential for the current to flow.

Thus, a current signal is obtained only if the tip during the sample contact constitutes a part of a closed electrical circuit. In this work, the nanoelectrical characterization was carried out without grounding the samples.

The TUNA results show that, even if the analyzed samples are not grounded, it is possible to detect electric current values that irrefutably prove the intrinsic electrical conductivity of the formulated nanocomposites.

The TUNA images were analyzed using the Bruker software Nanoscope Analysis 1.80 (Build R1.126200). To highlight the morphological peculiarities of the samples as well as their distribution within the polymeric matrix and their affinity with the epoxy domains, the nanocomposites underwent etching treatment before being investigated by TUNA. The etching reagent was prepared by stirring 1.0 g potassium permanganate in a solution mixture of 95 mL sulfuric acid (95–97%) and 48 mL orthophosphoric acid (85%). The filled resins were immersed into the fresh etching reagent at room temperature and held under agitation for 36 h. Subsequent washings were done using a cold mixture of two parts by volume of concentrated sulfuric acid and seven parts of water. Afterward the samples were washed again with 30% aqueous hydrogen peroxide to remove any manganese dioxide. The samples were finally washed with distilled water and kept under vacuum for 5 days before being subjected to morphological analysis.

References

1. Guadagno, L.; Raimondo, M.; Vertuccio, L.; Mauro, M.; Guerra, G.; Lafdi, K.; De Vivo, B.; Lamberti, P.; Spinelli, G.; Tucci, V. Optimization of graphene-based materials outperforming host epoxy matrices. *RSC Adv.* **2015**, *5*, 36969–36978. https://doi.org/10.1039/C5RA04558D.
2. Raimondo, M.; Guadagno, L.; Vertuccio, L.; Naddeo, C.; Barra, G.; Spinelli, G.; Lamberti, P.; Tucci, V.; Lafdi, K. Electrical conductivity of carbon nanofiber reinforced resins: Potentiality of Tunneling Atomic Force Microscopy (TUNA) technique. *Compos. Part B Eng.* **2018**, *143*, 148–160. https://doi.org/10.1016/j.compositesb.2018.02.005.
